# GENCODE reference annotation for the human and mouse genomes

**DOI:** 10.1093/nar/gky955

**Published:** 2018-10-24

**Authors:** Adam Frankish, Mark Diekhans, Anne-Maud Ferreira, Rory Johnson, Irwin Jungreis, Jane Loveland, Jonathan M Mudge, Cristina Sisu, James Wright, Joel Armstrong, If Barnes, Andrew Berry, Alexandra Bignell, Silvia Carbonell Sala, Jacqueline Chrast, Fiona Cunningham, Tomás Di Domenico, Sarah Donaldson, Ian T Fiddes, Carlos García Girón, Jose Manuel Gonzalez, Tiago Grego, Matthew Hardy, Thibaut Hourlier, Toby Hunt, Osagie G Izuogu, Julien Lagarde, Fergal J Martin, Laura Martínez, Shamika Mohanan, Paul Muir, Fabio C P Navarro, Anne Parker, Baikang Pei, Fernando Pozo, Magali Ruffier, Bianca M Schmitt, Eloise Stapleton, Marie-Marthe Suner, Irina Sycheva, Barbara Uszczynska-Ratajczak, Jinuri Xu, Andrew Yates, Daniel Zerbino, Yan Zhang, Bronwen Aken, Jyoti S Choudhary, Mark Gerstein, Roderic Guigó, Tim J P Hubbard, Manolis Kellis, Benedict Paten, Alexandre Reymond, Michael L Tress, Paul Flicek

**Affiliations:** 1 European Molecular Biology Laboratory, European Bioinformatics Institute, Wellcome Genome Campus, Hinxton, Cambridge CB10 1SD, UK; 2 UC Santa Cruz Genomics Institute, University of California, Santa Cruz, Santa Cruz, CA 95064, USA; 3 Center for Integrative Genomics, University of Lausanne, 1015 Lausanne, Switzerland; 4 Department of Medical Oncology, Inselspital, University Hospital, University of Bern, Bern, Switzerland; 5 Department of Biomedical Research (DBMR), University of Bern, Bern, Switzerland; 6 MIT Computer Science and Artificial Intelligence Laboratory, 32 Vasser St, Cambridge, MA 02139, USA; 7 Broad Institute of MIT and Harvard, 415 Main Street, Cambridge, MA 02142, USA; 8 Department of Molecular Biophysics and Biochemistry, Yale University, New Haven, CT 06520, USA; 9 Department of Bioscience, Brunel University London, Uxbridge UB8 3PH, UK; 10 Functional Proteomics, Division of Cancer Biology, Institute of Cancer Research, 123 Old Brompton Road, London SW7 3RP, UK; 11 Centre for Genomic Regulation (CRG), The Barcelona Institute for Science and Technology, Dr. Aiguader 88, Barcelona, E-08003 Catalonia, Spain; 12 Bioinformatics Unit, Spanish National Cancer Research Centre (CNIO), Madrid, Spain; 13 Department of Molecular, Cellular & Developmental Biology, Yale University, New Haven, CT 06520, USA; 14 Systems Biology Institute, Yale University, West Haven, CT 06516, USA; 15 Centre of New Technologies, University of Warsaw, Warsaw, Poland; 16 Department of Biomedical Informatics, College of Medicine, The Ohio State University, Columbus, OH 43210, USA; 17 Program in Computational Biology & Bioinformatics, Yale University, Bass 432, 266 Whitney Avenue, New Haven, CT 06520, USA; 18 Department of Computer Science, Yale University, Bass 432, 266 Whitney Avenue, New Haven, CT 06520, USA; 19 Universitat Pompeu Fabra (UPF), Barcelona, E-08003 Catalonia, Spain; 20 Department of Medical and Molecular Genetics, King's College London, Guys Hospital, Great Maze Pond, London SE1 9RT, UK

## Abstract

The accurate identification and description of the genes in the human and mouse genomes is a fundamental requirement for high quality analysis of data informing both genome biology and clinical genomics. Over the last 15 years, the GENCODE consortium has been producing reference quality gene annotations to provide this foundational resource. The GENCODE consortium includes both experimental and computational biology groups who work together to improve and extend the GENCODE gene annotation. Specifically, we generate primary data, create bioinformatics tools and provide analysis to support the work of expert manual gene annotators and automated gene annotation pipelines. In addition, manual and computational annotation workflows use any and all publicly available data and analysis, along with the research literature to identify and characterise gene loci to the highest standard. GENCODE gene annotations are accessible via the Ensembl and UCSC Genome Browsers, the Ensembl FTP site, Ensembl Biomart, Ensembl Perl and REST APIs as well as https://www.gencodegenes.org.

## INTRODUCTION

The GENCODE consortium produces foundational reference genome annotation for the human and mouse genomes as well as tools and data to maintain and improve these annotations. Our overall goal is to identify and classify, with high accuracy, all gene features in the human and mouse genomes based on defined biological evidence and to make these annotations freely available for the benefit of biomedical research and genome interpretation.

The GENCODE project was founded in 2003 as part of the pilot phase of the ENCODE project to provide reference quality manual gene annotation for the 30Mb (∼1%) of the reference human genome targeted by the ENCODE pilot (1–3). In 2007, we expanded our scope to the whole human genome as the ENCODE project did the same (4,5). In 2012, we began annotating the mouse reference genome to the same standards as human, while continuing to improve the existing gene annotation in both species via targeted reinvestigation of loci flagged by external users and internal QC pipelines. Today, the GENCODE consortium is a long-running partnership of manual annotation, computational biology and experimental groups including four of the founding groups (HAVANA, CRG, Yale and UCSC) and three groups that joined in 2007 (Ensembl, MIT and CNIO).

Our gene annotations are regularly released as the Ensembl/GENCODE gene sets. The gene sets are comprehensive and include protein-coding and non-coding loci including alternatively spliced isoforms and pseudogenes. To produce the annotations, we leverage computational and experimental methods to identify new genes and new transcript isoforms, directing manual annotation to regions requiring expert investigation. The Ensembl/GENCODE annotations are the default human and mouse annotation for the Ensembl project (6), while the UCSC Genome Browser (7) uses the human annotation as default and the mouse annotation as a secondary resource until the mouse clone-by-clone annotation is complete (see below). For each versioned release, the underlying genome annotation is exactly the same whether it is accessed at Ensembl, UCSC or https://genecodegenes.org, although there are minor differences in presentation associated with genome assembly patches and representation of the pseudoautosomal regions on the X and Y chromosomes. We also provide subsets of the annotation as described below. For simplicity, we will here refer to the annotation holistically as GENCODE.

GENCODE is the reference annotation of choice adopted by many large international consortia including ENCODE, GTEx (8), the International Cancer Genome Consortium (ICGC) (9), component projects of the International Human Epigenome Consortium (10), the 1000 Genomes Project, (11) the Exome Aggregation Consortium (EXAC) and Genome Aggregation Database (gnomAD) (12) and the Human Cell Atlas (HCA) (13).

## GENCODE ANNOTATION METHODS AND RESULTS

The GENCODE consortium annotates protein-coding genes, pseudogenes, long non-coding RNAs (lncRNAs) and small non-coding RNAs (sncRNAs). We define protein-coding genes as loci where the weight of available evidence supports the presence of a coding sequence (CDS). Evidence for a CDS may come from high-throughput experimental assays, the demonstration of physiological function in the research literature, the observation of homology to a known protein-coding gene, or the interpretation of evolutionary conservation data. Pseudogenes are sequences derived from protein-coding genes, containing disabling mutations such as in-frame stop codons, frameshifting indels, truncations or insertions, or for which there is no evidence of transcription. lncRNA genes are identified by a combination of transcriptional evidence and a lack of potential to be assigned as protein-coding. We do not absolutely require lncRNA genes to be longer than 200 bp, but very few annotated lncRNAs fall below this threshold, as we also require annotated lncRNAs to be free of secondary structures found in known functional sncRNAs. Currently, sncRNAs are almost entirely annotated by computational pipelines that use homology to known sncRNA sequences and predicted secondary structure to identify functional copies.

Our annotation processes use primary transcript and proteomics data, evolutionary conservation, computational methods and curated public databases such as UniProt (14). These data are integrated using a combination of expert manual annotators and computational methods to identify regions of the genome with genic potential, annotate the exon-intron structures of transcripts identified at the locus under investigation and assign a functional classification to both the individual transcript and the locus.

Broad functional classes (referred to as ‘biotypes’) of protein-coding, pseudogene, lncRNA and sncRNA are assigned as described above. More detailed functional categories are also added. For example, at the locus level we describe the provenance of pseudogenes as processed (derived via retrotransposition), unprocessed (defined by a genome duplication event) or unitary (arising from the lineage specific disruption of an ancestral protein-coding gene). At the transcript level we define transcripts belonging to protein-coding loci as protein-coding, nonsense mediated decay (NMD) (containing a premature stop codon believed likely to lead to the transcript being targeted by the nonsense-mediated decay pathway) or retained intron (containing sequence that is intronic in other transcripts from the locus). Following the structural and functional classification of transcripts, a subset of GENCODE annotation is subject to targeted experimental validation as described below to ensure consistent high quality of the gene annotation.

To cater for a variety of use cases, we create a number of annotation sets. Examples of these are our ‘GENCODE comprehensive’ and ‘GENCODE basic’ gene sets. GENCODE comprehensive includes the complete set of annotations including partial transcripts (i.e. transcripts that are not full length, but represent a unique splice form based on available evidence) and biotypes such as NMD. GENCODE basic is a subset of GENCODE comprehensive that contains only transcripts with full-length CDS. For non-coding loci, GENCODE basic includes the smallest number of transcripts that cover 80% of the exonic features, while ensuring all loci are represented by at least 1 transcript. Computational methods add additional information. For example, APPRIS, described in more detail below, identifies the most likely functional translations at protein-coding loci and TSL (transcript support level) calculates the amount and quality of supporting evidence for each transcript.

### Manual annotation

The GENCODE gene set is created by merging the results of manual and computational gene annotation methods. Manual gene annotation has two major modes of operation: clone-by-clone and targeted annotation. ‘Clone-by-clone’ annotation involves ‘walking’ across a genomic region, investigating the sequence, aligned expression data and computational predictions for each BAC clone. In doing so, an expert annotator investigates all possible genic features and considers all possible annotations and biotypes simultaneously. We believe this approach carries substantial advantages. For example, the decision to annotate a locus as protein-coding or pseudogenic benefits from being able to weigh both possibilities in light of all available evidence. This process helps prevent false positive and false negative misclassifications. Targeted annotation is designed to answer specific questions such as ‘is there an unannotated protein-coding gene in this position?’ Ranked target lists are generated by computational analysis based, for example, on transcriptomic data, shotgun proteomic data or conservation measures. Over the last two years mouse annotation has been dominated by the clone-by-clone approach while the human genome has been refined entirely via targeted reannotation except for the annotation of human assembly patches and haplotypes released by the Genome Reference Consortium (15), which take a clone-by-clone approach.

Over the last two years, we have focused on two broad areas: completing the first pass manual annotation across the entire mouse reference genome and a dedicated effort to improve the annotation of protein-coding genes in human and mouse.

We have completed the annotation of novel protein-coding genes, lncRNAs and pseudogenes, plus QC and updating previous annotation where necessary for mouse chromosomes 9, 10, 11, 12, 13, 14, 15, 16 and 17. These updates bring the fraction of the mouse genome with completed first pass manual annotation to approximately 97%. In addition, we have continued to work with the NCBI and Mouse Genome Informatics project at the Jackson Laboratory to resolve annotation differences for protein-coding, pseudogene and lncRNA loci. For protein-coding genes this is under the umbrella of the Consensus Coding Sequence (CCDS) project (16).

We have also manually investigated unannotated regions of high protein-coding potential identified by whole genome analysis using PhyloCSF (17) (a tool described in more detail below). In human, this led to the addition of 144 novel protein-coding genes and 271 pseudogenes (of which 42 were unitary pseudogenes). In mouse, we annotated orthologous loci for all but 11 of the 144 human protein-coding genes. We have also revisited the annotation of all olfactory receptor loci in both human and mouse, using RNAseq data to define 5′ and 3′ UTR sequences for ∼1400 loci. In human we have also targeted a ‘deep dive’ manual reannotation of genes on clinical panels for paediatric neurological disorders to identify missing functional alternative splicing. Incorporating second and third generation transcriptomic data, we reannotated ∼190 genes and added more than 3600 alternatively spliced transcripts, including ∼1400 entirely novel exons and an additional ∼30kb of CDS. We have also completed an effort to capture all recently described unannotated microexons (18) into GENCODE, and further added an additional 146 novel microexons mined from public SLRseq data (19).

As part of the CCDS collaboration with RefSeq, we have checked a large subset of human loci where there was disagreement over gene biotype. Similarly, we have checked all UniProt manually annotated and reviewed (i.e. Swiss-Prot) accessions that lack an equivalent in GENCODE. As a result, we added 32 novel protein-coding loci to GENCODE and rejected more than 200 putative coding loci. Finally, we are manually reviewing genes previously annotated as protein-coding, but with weak or no support based on a method incorporating UniProt, APPRIS, PhyloCSF, Ensembl comparative genomics, RNA-seq, mass spectrometry and variation data (20,21). Of the 821 loci investigated to date, 54 have had their coding status removed while a further 110 potentially dubious cases remain under review.

**Figure 1.**
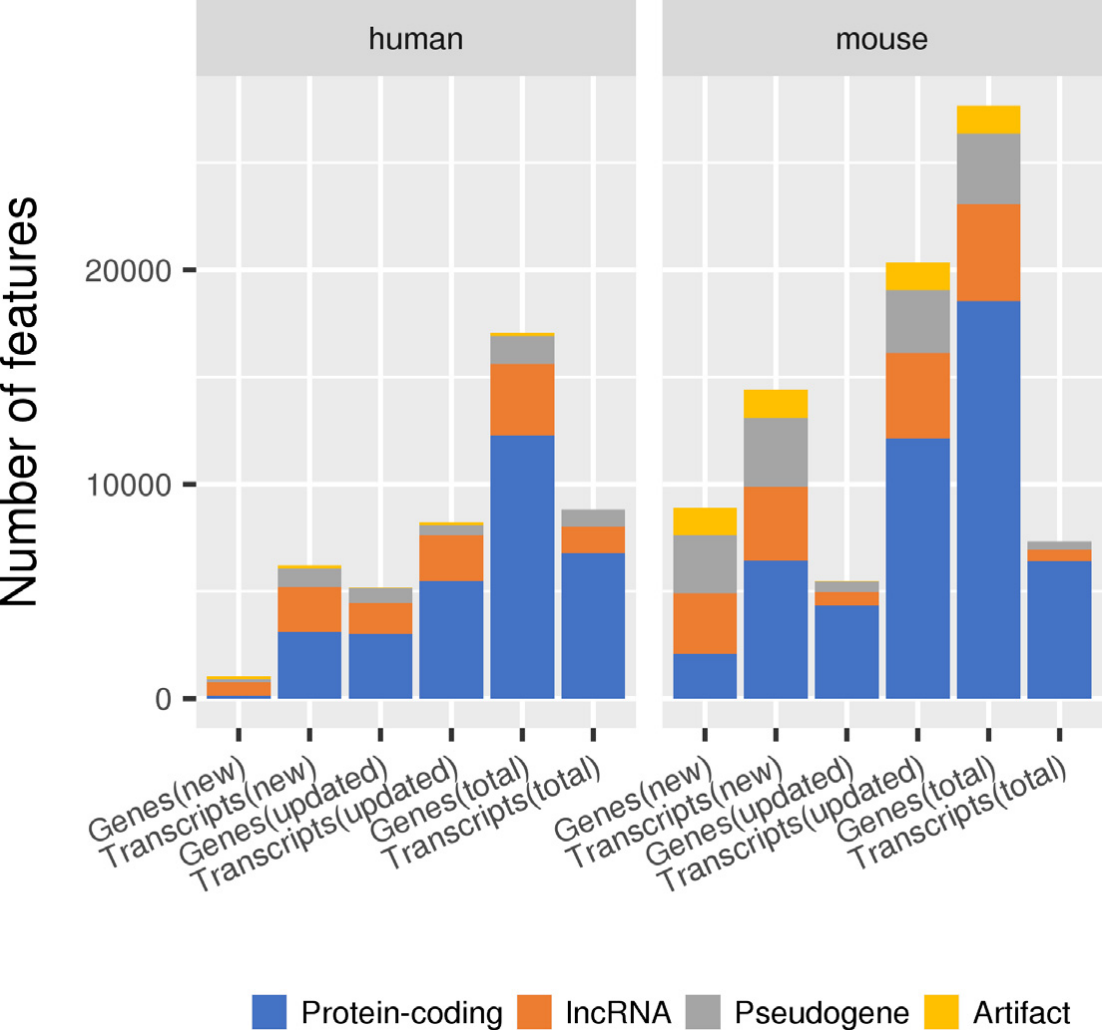
New and updated manually annotated genes and transcripts from July 2016 to June 2018. For both human (left) and mouse (right) the numbers of completely new genes and transcripts, updated genes and transcripts and the total number of manually added or edited genes and transcripts for each of four broad categories of annotation. A new gene annotation can represent a completely *de novo* locus with no overlap with pre-existing annotation or the reclassification of an existing complex locus into multiple loci to better represent the biology of the locus inferred from transcriptomic and/or proteomic data. A new transcript represents the annotation of a unique exon-intron structure, including novel alternative splicing at an annotated locus. Updated genes and transcripts represent pre-existing loci or transcript models that have been edited to improve the representation of biotype (e.g. changed from lncRNA to protein-coding) or structure (e.g. by extension, addition of novel exons).

The approach taken reflects in the kinds of updates captured in the annotation. For example, the targeted reannotation in human leads to the annotation of few novel protein-coding loci but many novel transcripts at updated protein-coding and lncRNA loci. Conversely, in mouse the emphasis on clone-by-clone annotation identifies many more novel loci and transcripts across a broader range of biotypes (Figure 1).

### Computational annotation of small RNAs

We annotate small non-coding RNAs (sncRNAs) using a variety of mechanisms. Specifically, miRNA annotations are imported directly from miRBase (22), while tRNAs are identified *ab initio* using tRNAScan-SE (23) although they are not included directly in the gene set. For other classes of sncRNA, including small nucleolar RNAs (snoRNAs), small nuclear RNAs (snRNAs) and small Cajal body-specific RNAs (scaRNAs), we use a homology-based, computational pipeline (24), which first compares sequences of known RNA families in Rfam (25) to the genome using BLAST (26). This initial step reduces the genomic search space and excludes sequences with sub-optimal alignments to the genome. We define putative sncRNA models after clustering top BLAST hits and evaluating these predictions by performing sequence and structure searches against covariance models in the Infernal suite of tools (27).

### Pseudogenes

Pseudogene annotations across 18 mouse strains were generated using a combination of manual annotation liftover and computational methods. Additionally, we were able to annotate 88 new human and 131 new mouse unitary pseudogenes relative to each other. Amongst the strains we find roughly 20 unitary pseudogenes per strain. We identified nearly 3000 ancestral pseudogenes conserved across all strains. Meanwhile, ∼20% of the pseudogenes in each strain are strain specific. In line with previous results in human, 15% of pseudogenes exhibit transcriptional activity (bioRxiv: https://doi.org/10.1101/386656).

## EXPERIMENTAL ANNOTATION APPROACHES

### lncRNA annotation using capture long Seq

Determining the precise boundaries and the exonic structure of low abundant transcripts, such as lncRNAs is challenging. We previously showed that 3′ and 5′ boundaries of lncRNAs annotated in GENCODE V7 (April 2011) were less supported by CAGE and PET tags than those of protein-coding genes, even when accounting for differences in expression (28). Methods to assemble transcript sequences from short sequence reads have also been shown to produce poor results when used to resolve the exonic structure of lncRNAs (29,30). To improve lncRNA annotation, we developed the RNA Capture Long Seq (CLS) method (31). Here, probes are first designed against targeted lncRNAs (or suspected, unannotated lncRNA loci). Full-length cDNAs generated from diverse cell types were captured, resulting in cDNA libraries that are highly enriched for the targeted lncRNAs. Libraries were then sequenced using long-read sequencing technologies (31,32). Our initial efforts created a comprehensive capture library targeting the set of intergenic GENCODE lncRNAs in human and mouse, and used it in a set of matched human and mouse tissues (31). This resulted in novel lncRNA transcripts at 3574 loci in human, and 561 in mouse. The long length of the transcript sequences obtained, often correspond to complete 5′-to-3′ RNA molecules, substantially informed manual annotation. Indeed, CLS produces near manual-quality full-length transcript models at high-throughput scales (32). Our current efforts are to include samples across a more diverse panel of tissues such as fetal timepoints.

### Proteomics

Proteomic mass spectrometry datasets are a powerful resource contributing to the validation and annotation of protein-coding genes and transcripts. In GENCODE, we use proteomics data as an additional layer of evidence when defining the structure and protein-coding potential of a genomic locus. We apply strict criteria to the peptide evidences we consider from mass spectrometry datasets (33–35) to minimize the incorporation of false positive and ambiguous or variant peptide species. In highly curated genomes such as human, the contribution from mass spectrometry experiments requires considerable scale of data and effort, with correspondingly small returns. Our experimental efforts in GENCODE incorporate targeted proteomics experiments, specific experimental designs and synthetically generated peptides to find these elusive protein-coding genes.

### Annotation validation and RACEseq

We used RT-PCR amplification followed by highly multiplexed sequencing readout (36) to assess the quality of the annotations. This method evaluates low confidence transcribed loci (novel or putative). Splice site loci were systematically experimentally tested in eight tissues (brain, heart, kidney, liver, lung, spleen, skeletal muscle, and testis) by RT-PCR-seq (36). From human GENCODE versions 3 to 19, a total of 18 132 splice junctions were analyzed and experimentally tested. Seventy eight percent of all assessed junctions were confirmed through experimental validation. Similar to the human annotation, we assessed the quality of the mouse annotation. A total of 3956 splice junctions from GENCODE versions M2 and M4 were tested with a validation rate of 53%. Finally, to assess the completeness of the annotations we amplified and sequenced the transcripts of 527 deeply annotated human protein-coding genes, which are routinely used for diagnostic tests by the UK Genetic Testing Network (UKGTN). We performed 5′- and 3′- nested- RACEs in seven different tissues (brain, testis, heart, kidney, liver, lung, and spleen) followed by long-read sequencing, which revealed 10 380 novel splice junction candidates.

## GENCODE ANNOTATION TOOLS

### Comparative annotation toolkit

We developed the Comparative Annotation Toolkit (CAT) (37) to leverage the GENCODE annotations of mouse and human to annotate laboratory mouse strains (38) and great apes (39,40). CAT uses whole genome alignments from Cactus (41) to project GENCODE annotations from mouse or human to related species, and then performs a variety of filtering and clean-up steps to generate a high quality annotation set for these other genomes. The GENCODE M11 mouse annotation was used with CAT to annotate 16 laboratory mouse strains, and these annotations are available in Ensembl. Over 20 000 protein-coding and 12 000 non-coding genes were comparatively annotated in each lab strain. Novel gene predictions using Comparative Augustus (42) also found an average of 22 new loci in classical strains, including the discovery of the gene *Efcab3-like* in the reference mouse, which was included in subsequent GENCODE releases. Additionally, the GENCODE 27 (August 2017) human annotation set was used to annotate chimpanzee, gorilla and orangutan, and these annotations were incorporated into Genbank, with over 19 000 protein-coding and 36,000 non-coding genes comparatively annotated in all of the great apes.

### APPRIS

The APPRIS Database (http://appris-tools.org) (43) was developed to provide annotations for alternative splice variants. APPRIS also determines principal splice isoforms based on cross-species conservation and the conservation of protein structure and function. Most coding genes have a single dominant protein isoform and this main isoform is almost always the APPRIS principal isoform (44).

APPRIS maintains up-to-date annotations for the GENCODE and RefSeq reference sets and has been extended to the UniProtKB proteome and to six model species as well as human and mouse (45). Technical improvements include incremental improvements to the core modules that make up the APPRIS pipeline, the implementation of a UCSC Track Hub to make annotation access easier, and Docker images to allow the execution of the annotation pipeline (45).

APPRIS is an integral part of the pipeline for the prediction of potential non-coding genes (20). For the GENCODE 27 (August 2017) human annotation the completed pipeline flagged 2432 genes.

### PhyloCSF

Comparative genomics is one of the most powerful tools available for distinguishing protein-coding genomic regions. Previously, we developed PhyloCSF to support annotation of coding sequences based on the alignment of multiple genome sequences (17). As described above, we combine whole-genome PhyloCSF data with experimental evidence and expert manual annotation to detect novel coding sequences. The workflow begins with PhyloCSF scores computed on every codon in the human genome in each of the six reading frames; applies a Hidden Markov Model to these scores to find candidate coding intervals; excludes intervals previously annotated as coding or pseudogene, or antisense to such intervals, as well as very short intervals; and uses a Support Vector Machine to prioritize the resulting ‘Novel PhyloCSF Regions’. We have created publicly available PhyloCSF track hubs for viewing the whole-genome PhyloCSF data and novel PhyloCSF Regions from human and mouse in the UCSC and Ensembl genome browsers.

### Pseudopipe

Pseudopipe identifies and annotates pseudogenes across the genome (46). It takes as input an organism's protein-coding gene set and searches for homology across the genome using BLAST. Hits overlapping functional genes are removed and the remaining hits are then assembled into pseudogene annotations. Each annotation is also assigned a parent gene, the functional paralog that gave rise to the pseudogene, as well as a biotype (processed, duplicated, or ambiguous). Unitary pseudogenes are also identified via Pseudopipe by using a different organism's protein-coding gene set as the input. We inform our annotation with results from Retrofinder (47) and RCPedia (48). In addition to our core annotation files, further information is available at http://www.pseudogene.org. These computational annotations are then combined with manual annotations in order to produce the full pseudogene complement. Pseudogene annotations are given a confidence level based on the intersection with manual annotations. Annotations detected by both the computational pipelines and manual annotators are assigned level 1, those only detected by manual annotators are given level 2, and the consensus annotations detected by PseudoPipe and RetroFinder are given level 3 and made available in a separate annotation file at https://www.gencodegenes.org.

## DATA ACCESS

**Figure 2.**
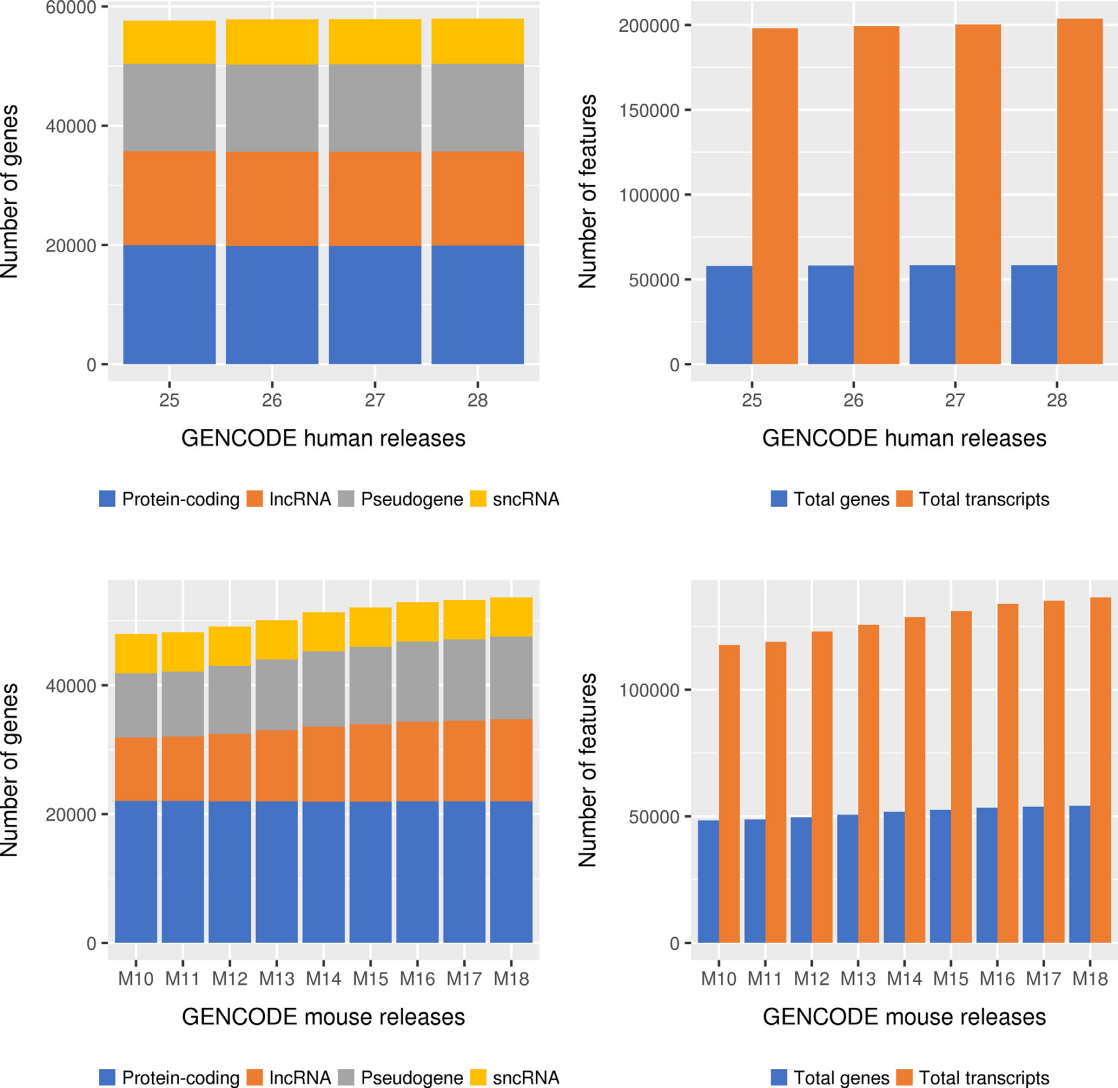
Annotation statistics for human and mouse GENCODE releases from July 2016 to June 2018, encompassing human releases GENCODE 25–28 and mouse releases M10 to M18. The panels on the left show the total number of genes by broad biotype (protein-coding, lncRNA, pseudogene and sncRNA) for each release for human and mouse respectively and panels on the right show the total numbers of genes and transcripts of all biotypes.

Versioned GENCODE gene sets are currently released approximately four times a year for mouse and twice a year for human. This asymmetric update pattern reflects the fact that the first pass of the human annotation was completed in GENCODE 15 (January 2013), while the mouse first pass is approaching completion (expected for GENCODE M20) and therefore has been the subject of more intensive annotation. The most recent release of the human geneset is GENCODE 29 (October 2018), while the most recent mouse update is GENCODE M19 (October 2018). Each release incorporates the continuous updates arising from expert manual annotation. Figure 2 shows the increase in the numbers of genes and transcripts in human and mouse GENCODE releases over the past two years. The human genesets look relatively static, although headline figures do not capture updates made to existing annotation and the balancing effect of both adding and removing loci during a release cycle. In mouse however, there is clear growth in the numbers of both genes and transcripts driven predominantly by the addition of lncRNAs and pseudogenes.

Extensive data resources for current and archival GENCODE releases are available at https://www.gencodegenes.org. As described above, the GENCODE gene sets are available as default in the Ensembl genome browser and also accessible via the UCSC genome browser. Other interfaces include the Ensembl FTP site (ftp://ftp.ensembl.org/pub/), which includes gene sets in GFF3, Genbank and GTF formats and full download of the complete Ensembl databases. More complex and customizable gene set queries can be created via the Ensembl Biomart (https://www.ensembl.org/biomart/).

Programmatic access to the GENCODE gene sets is possible via the extensive Ensembl Perl API and the language-agnostic Ensembl REST API. Programmatic access facilitates advanced genome-wide analysis such as retrieval of supporting features and associated gene trees. Examples of REST endpoint usage and starter scripts in different languages are at https://rest.ensembl.org.

GENCODE has been created exclusively on the GRCh38 human assembly since GENCODE 20 (August 2014). However, versions of selected releases since then that have been projection mapped from GRCh38 to GRCh37 are available at UCSC and from https://www.gencodegenes.org. Referred to as the ‘lift37’ annotation set, these data help identify genes where the annotations may have changed between GRCh37 and GRCh38. Due to the difficulty to generate accurate projections, the ‘lift37’ annotation set is not considered official reference annotation and only minimal support is available.

We welcome questions and feedback from the community directly via the helpdesks at https://www.gencodegenes.org, Ensembl and UCSC. In addition, the Ensembl and UCSC outreach activities annually reach thousands of researchers via workshops at institutions and meetings, web-based training forums and ‘how-to’ guides focused on using the genome browsers and making best use of their features and data.

## CONCLUSION

The GENCODE consortium continues to improve the quality of the reference gene annotation in human and mouse. We have integrated cutting-edge developments in the technology and scientific understanding of genome biology into our annotation workflows to improve the representation of existing loci and extend annotation coverage via the addition of entirely novel loci and alternatively spliced transcripts. While the high quality of our existing transcript annotation is extensively supported by both public data and data generated within the consortium, the abundance of evidence from new transcriptomic and proteomic datasets makes it clear that they are not yet complete.
